# Multi-drug resistant gram-negative bacterial pneumonia: etiology, risk factors, and drug resistance patterns

**DOI:** 10.1186/s41479-022-00096-z

**Published:** 2022-05-05

**Authors:** Muluneh Assefa

**Affiliations:** Department of Medical Microbiology, School of Biomedical and Laboratory Sciences, College of Medicine and Health Sciences, University of Gondar, P.O. Box 196, Gondar, Ethiopia

**Keywords:** Pneumonia, Multi-drug resistance, Gram-negative bacteria

## Abstract

Bacterial pneumonia is one of the most serious public health issues owing to its medical and economic costs, which result in increased morbidity and mortality in people of all ages around the world. Furthermore, antimicrobial resistance has risen over time, and the advent of multi-drug resistance in GNB complicates therapy and has a detrimental impact on patient outcomes. The current review aimed to summarize bacterial pneumonia with an emphasis on gram-negative etiology, pathogenesis, risk factors, resistance mechanisms, treatment updates, and vaccine concerns to tackle the problem before it causes a serious consequence. In conclusion, the global prevalence of GNB in CAP was reported 49.7% to 83.1%, whereas in VAP patients ranged between 76.13% to 95.3%. The most commonly reported MDR-GNB causes of pneumonia were *A. baumannii, K. pneumoniae, *and* P. aeruginosa, *with* A. baumannii *isolated particularly in VAP patients and the elderly*.* In most studies, ampicillin, tetracyclines, amoxicillin-clavulanic acid, cephalosporins, and carbapenems were shown to be highly resistant. Prior MDR-GNB infection, older age, previous use of broad-spectrum antibiotics, high frequency of local antibiotic resistance, prolonged hospital stays, ICU admission, mechanical ventilation, and immunosuppression are associated with the MDR-GNB colonization. *S. maltophilia* was reported as a severe cause of HAP/VAP in patients with mechanically ventilated and having hematologic malignancy due to its ability of biofilm formation, site adhesion in respiratory devices, and its intrinsic and acquired drug resistance mechanisms. Effective combination therapies targeting PDR strains and drug-resistant genes, antibiofilm agents, gene-based vaccinations, and pathogen-specific lymphocytes should be developed in the future.

## Introduction

Pneumonia is an acute inflammation and consolidation of lung tissue due to infectious agents such as bacteria, viruses, fungi, and parasites [1]. Bacterial pneumonia is an inflammation of one or two lobes of the lung due to bacterial infection [2]. Based on how the infection is acquired, pneumonia can be classified into community-acquired pneumonia (CAP) and hospital-acquired pneumonia (HAP) [3]. According to Temesgen and his colleague’s report in 2019, CAP is an infection of the lung parenchyma that is not acquired from a hospital or health care facility [4]. Hospital-acquired pneumonia is defined as pneumonia that occurs after 48 h or more of hospital admission, and if associated with mechanical ventilation, it is termed ventilator-associated pneumonia (VAP) [5]. The global burden of diseases, injuries, and risk factors study in 2017 reported that lower respiratory tract infections (LRTIs), including bacterial pneumonia, cause nearly 2.56 million deaths among all age groups, making LRTIs the fifth leading cause of mortality with higher fatalities in Sub-Saharan Africa, South Asia, and Southeast Asia [6, 7].

Bacterial pneumonia can spread via aspiration, inhalation, or bloodstream spread of pathogenic bacteria [8]. Pneumonia is a result of an infection caused by the immune system’s inability to clear a pathogen from the lower airway and alveoli. This leads to the bronchioles and alveoli being filled with inflammatory exudates of leukocytes and fluid. This results in decreased carbon dioxide and oxygen exchange between the blood and the lungs, causing respiratory scarcities and symptoms such as cough, sputum production, dyspnea, chest pain, and respiratory dysfunction and/or shock in severe cases [9, 10]. According to several studies, age, incomplete or inadequate vaccination, indoor environmental exposure, medical conditions such as asthma, diabetes, heart disease, treatment-induced cytopenias in cancer, long-term hospitalization, malnutrition, immunosuppression, smoking, alcohol consumption, poor dental hygiene, contact with contaminated hospital equipment, previous exposure to antibiotics, and the presence of viral infections that compromise the respiratory tract that results in secondary bacterial colonization and infection are all important risk factors for disease development [11–15].

Studies documented that *Streptococcus pneumoniae*, *Staphylococcus aureus*, *Klebsiella pneumoniae*, *Haemophilus influenzae*, *Pseudomonas aeruginosa, Moraxella catarrhalis,* and *Escherichia coli* were the most frequent causes of typical pneumonia, whereas atypical pneumonia is mostly caused by *Legionella pneumophila*, *Chlamydia pneumoniae,* and *Mycoplasma pneumoniae. Even though S. pneumoniae* is the most prevalent cause of CAP in all age groups around the world, gram-negative bacteria (GNB) such as *K. pneumoniae, Acinetobacter baumannii, P. aeruginosa,* and *E. coli* are commonly related to HAP [3, 16, 17]. Antibiotic resistance is increasingly being recognized as a major worldwide health concern resulting from antibiotic overuse and improper administration [18]. Nowadays, pneumonia caused by multidrug-resistant gram-negative bacteria (MDR-GNB) is growing more common and has a detrimental impact on patient outcomes, indicating a shift in infection trends to GNB and their rapid dissemination, particularly in the hospital settings [19–23]. To address this, the current review is intended to provide a summary of the findings on bacterial pneumonia focusing on gram-negative etiology, their pathogenesis, mechanisms of resistance to antibiotics, risk factors, diagnostic challenges and advancements, updates on treatment options, and vaccine issues which enables concerned bodies to tackle the problem before it causes a serious consequence.

### Epidemiology and burden

Bacterial pneumonia continues to be one of the most serious public health problems due to its medical and economic burden. Both CAP and HAP increase morbidity and death in people of all ages around the world [24, 25]. Community-acquired pneumonia is the sixth leading cause of death in people aged 65 and above worldwide. In developed countries, the estimated incidence of CAP is 0.2 to 1.1% in adults, and the mortality is 2 to 14% [26]. A population-based study by Bjamason et al. [27] reported that CAP requiring hospitalization was 2.7 cases per 1000 adults annually. The incidence is higher in children under the age of four and people over the age of 60, with more than 12 cases per 1000 people, but in adults, the rate is usually 5.2 to 7.1 cases per 1000 persons per year [28]. Childhood mortality and adult hospitalization due to pneumonia remain increasing in low and middle-income countries. The frequency has increased in the elderly because of physiological changes linked to the progressive dysfunction of the respiratory tract and/or weakened immunity supported by 72.6% GNB prevalence in elderly patients with CAP from China [26, 29].

According to hospital-based studies in Africa, CAP is linked to a 6 to 15% increase in adult inpatient hospital mortality [30]. Community-acquired pneumonia is the most common cause of adult hospitalization and mortality, accounting for 10% in Kenya [31], 11.9% in Nigeria [32], 17% in Ethiopia [33], and 51,000 admissions with 10,000 deaths in Malawi [34] each year. Epidemiological reports from Sub-Saharan Africa also revealed high rates of morbidity and mortality from the disease, with an estimated 4 million cases and 200,000 deaths per year [35, 36]. Pneumonia is one of the leading causes of death among Ethiopian children under the age of five, accounting for 28% of all deaths [33]. Previous reports in Africa showed GNB prevalence in CAP with 49.7% to 56.7% in Ethiopia [4, 37, 38], 83.1% in Tanzania [39], and 76.2% in Sudan [40].

Hospital-acquired pneumonia is the second most common nosocomial infection in the world, affecting 0.5 to 1.7% of hospitalized patients. It is also the leading cause of death among all nosocomial infections [41]. The incidence of HAP ranges from 5 to 20 or more cases per 1000 hospital admission [42]. A study in Ethiopia by Tassew et al. [43] in hospitalized patients reported HAP as the common type of infection, accounting for 24.7%. Ventilator-associated pneumonia is the most common nosocomial infection in the intensive care unit (ICU), accounting for 25% of all ICU infections [44]. A Kenyan study reported VAP prevalence of 54.4% among 92 patients with clinical pulmonary infection [45]. In a large cohort study, both HAP and VAP in ICU patients were associated with 82% and a 38% increase in the risk of 30-day mortality, respectively [46]. Gram-negative bacteria are responsible for most bacterial causes of HAP/VAP (50–80%) [47]. In the US and Europe, HAP and VAP due to GNB among ICU patients were 61.5 and 76.1%, respectively [48]. A study conducted on Egyptian children revealed that GNB was more prevalent in HAP and VAP (91.67 and 87.8%, respectively) [49]. In Iran, GNB was obtained in 72.2 and 84.6% of HAP and VAP, respectively [50]. Feng et al. [51] reported a 14.5% mortality rate of HAP related to GNB in a retrospective, single-center analysis study in China. Moreover, MDR, extensively drug-resistant (XDR), and pan-drug resistant (PDR) bacteria, especially GNB, are increasingly isolated in HAP and VAP and are associated with mortality rates over 50% [52].

### Etiology

Recently, several studies on the bacterial cause of pneumonia have been published. Studies reported the study period, the number of study participants, age category, pneumonia type (CAP, HAP, and VAP), specimen sources, as well as studies performed culture and antimicrobial susceptibility testing have been included and summarized (Tables 1 and 2). In studies conducted on the etiology of CAP, GNB was found to be present 49.7 to 83.1% of the time, with common etiologic agents of *K. pneumoniae, P. aeruginosa,* and *E. coli* [4, 26, 37–40] (Table 1). The very high prevalence of GNB in some studies is related to variation in the sample size, geographic location, study period, study population, and specimen contamination of respiratory flora. Ventilator-associated pneumonia caused by multidrug-resistant GNB has emerged as a significant and intractable clinical problem [58]. Studies in VAP patients reported that GNB prevalence between 76.13 to 95.3% with highly MDR *P. aeruginosa, and A. baumannii* strains [53–57] (Table 2).

**Table 1:** Summary of the isolation and drug resistance profile of GNB in CAP

Country	Study year	Participants, age group, pneumonia category	Specimen	GNB (%) in culture	Frequently isolated GNB (%)	Decreasing order of resistance, MDR (%)	References
Tanzania	2015	353, adult, CAP	Sputum	83.1%	*K. pneumoniae* (29.9%) and *P. aeruginosa* (11.7%)	*K. pneumoniae*: AMP > AMC > CRO *P. aeruginosa*: AMP/SXT/AMC > CRO > CIP	[39]
China	2016 to 2017	176, older (> 60 years), CAP	Sputum	72.6%	*K. pneumoniae* (27.4%), *E. coli* (17.9%), and *P. aeruginosa* (10.3%)	*K. pneumoniae*: AMP > CXM > PIP, 25.0% ESBL *E. coli*: AMP > CXM > CTX > PIP, 42.9% ESBL *P. aeruginosa*: MIN > SXT > CXM	[26]
Sudan	2017	100, 16 to 60 years, CAP	Sputum	76.2%	*K. pneumoniae* (42.8%) and *P. aeruginosa* (30.9%)	*K. pneumoniae*: CAZ/PEP/CRO/CTX, 16.7% ESBL *P. aeruginosa*: PIP > CL > IMP	[40]
Ethiopia	2018	414, adult, CAP	Sputum	49.7%	*K. pneumoniae* (18.0%) and *P. aeruginosa* (11.4%)	*K. pneumoniae*: AMP/TE > AMC > SXT > C/DO > CN > CIP, 100% *P. aeruginosa*: CN > CRO > CIP > PIP > CAZ, 42.1%	[4]
Ethiopia	2020	406, ≥ 5 years, CAP	Sputum	56.7%	*K. pneumoniae* (28.0%) and *P. aeruginosa* (14.0%)	*K. pneumoniae*: AMP > TE > SXT/AMC > C > AK > CAZ > PZP/CXM > CIP, 97.7% *P. aeruginosa*: CAZ > CN > TE/PEP/AK > CIP, 45.5%	[37]
Ethiopia	2021	312, adult, CAP	Sputum	53.2%	*K. pneumoniae* (31.0%) and *E. coli* (20.7%)	*K. pneumoniae*: AMP > AMC > SXT > TE/DO, 94.9% *E. coli*: AMP > TE > DO > SXT > AMC, 93.8%	[38]

*CAP* community-acquired pneumonia, *HAP* hospital-acquired pneumonia, *VAP* ventilator-associated pneumonia, *ESBL* extended-spectrum beta-lactamase, *AMP* ampicillin, *AMC* amoxicillin/clavulanate, *TOB* tobramycin, *TZP* piperacillin/tazobactam, *CRO* ceftriaxone, *AK* amikacin, *CXM* cefuroxime, *CTX* cefotaxime, *CIP* ciprofloxacin, *SXT* trimethoprim-sulfamethoxazole, *CL* colistin, *CN* gentamicin, *IMP* imipenem, *CAZ* ceftazidime, *TE* tetracycline, *C* chloramphenicol, *PEP* cefepime, *MER* meropenem, *MIN* minocycline, *APS* ampicillin/sulbactam, *TLV* ticarcillin/clavulanate, *LEV* levofloxacin, *AZT* aztreonam, *DO* doxycycline

**Table 2:** Summary of the isolation and drug resistance profile of GNB in HAP/VAP

Country	Study year	Participants, age group, pneumonia category	Specimen	GNB (%) in culture	Frequently isolated GNB (%)	Decreasing order of resistance, MDR (%)	References
India	2012 to 2014	87, all age, VAP	Tracheal aspirates	88.3%	*P. aeruginosa* (38.3%), *A. baumannii* (15.6%), and *K. pneumoniae* (14.3%)	*P. aeruginosa*: PEP > CAZ > CN, 73.1% *A. baumannii*: CXT > CTX > PEP, 83.3% *K. pneumoniae*: CXT > CTX > PEP, 72.2%	[53]
Egypt	2014 to 2015	153, all age, VAP	Tracheal aspirates	87.1%	*K. pneumoniae* (36.9%), *E. coli* (21.04%), *A. baumannii* (14.95%), and *P. aeruginosa* (14.16%)	*K. pneumoniae*: CRO > PEP > AZT > CIP/TE > SXT > TZP > CN > AK > IMP, 100% *E. coli*: CTR > AZT/PEP > TE > CIP > SXT > CN > TZP > AK > IMP, 98% *A. baumannii*: CAZ/CTR/PEP/IMP/LEV/MER/PIP > AK > SXT > CN, 100% *P. aeruginosa*: CAR/CRO > CAZ > SXT/PIP/CIP/CN > AK/C > IMP/TZP, 84.84%	[54]
Bangladesh	2015 to 2016	51, all age, VAP	Tracheal aspirates	76.13%	*A. baumannii* (37.5%), *K. pneumoniae* (22.7%) and *P. aeruginosa* (13.6%)	*A. baumannii*: CRO/CAZ/CTX > AZT/PEP > AK/CN/COT > CIP > IMP > PZP *K. pneumoniae*: CRO/CAZ/CTX/PEP > CPR > SXT > CN > AK *P. aeruginosa*: PEP/CTX/CRO > CN/SXT/IMP > CAZ/CIP	[55]
Vietnam	2017 to 2018	103, 32 to 94 years old, HAP	Sputum and bronchoalveolar lavage	95.3%	*A. baumannii* (47.5%), *K. pneumoniae* (16.2%) and *P. aeruginosa* (12.1%)	*A. baumannii*: APS/PEP/CTX/CAZ/CRO/MER/CIP/LEV > PZP > TLV > TOB, 100% *K. pneumoniae*: AMP > TOB/PEP/CAZ/SXT > TLV/CRO > AK/CN, 72.7% *P. aeruginosa*: CTX > CRO/TLV/CN > TOB/PEP/CIP > AK, 92.3%	[56]
Tanzania	2019 to 2020	269, adult, VAP	Bronchial aspirate	80.1%	*P. aeruginosa* (24.7%),*K. pneumoniae* (19.8%), and *E. coli* (12.4%)	*P. aeruginosa*: CAZ/CXM/AZT/CIP > CN, 73.9% *K. pneumoniae*: CRO > AMC > CIP > PIP, 76.4% *E. coli*: CN > CRO > CAZ, 70%	[57]

*CAP* community-acquired pneumonia, *HAP* hospital-acquired pneumonia, *VAP* ventilator-associated pneumonia, *ESBL* extended-spectrum beta-lactamase, *AMP* ampicillin, *AMC* amoxicillin/clavulanate, *TOB* tobramycin, *TZP* piperacillin/tazobactam, *CRO* ceftriaxone, *AK* amikacin, *CXM* cefuroxime, *CTX* cefotaxime, *CIP* ciprofloxacin, *SXT* trimethoprim-sulfamethoxazole, *CL* colistin, *CN* gentamicin, *IMP* imipenem, *CAZ* ceftazidime, *TE* tetracycline, *C* chloramphenicol, *PEP* cefepime, *MER* meropenem, *MIN* minocycline, *APS* ampicillin/sulbactam, *TLV* ticarcillin/clavulanate, *LEV* levofloxacin, *AZT* aztreonam, *DO* doxycycline

### Drug resistance patterns of GNB

Drug resistance in GNB varied from place to place and studies reported high drug resistance in elderly patients and all age groups in VAP. This may be due to the increased exposure to antibiotics in the elderly and the high frequency of MDR-GNB in the hospital areas related to VAP (Tables 1 and 2). In a study of drug resistance analysis on older CAP outpatient reports, ESBL producing strains were detected in *E. coli* (42.9%) and *K. pneumoniae* (25.0%) [26]. A study in Ethiopian adult CAP patients showed MDR prevalence of 100% in *K. pneumoniae, P. vulgaris,* and *H. influenzae*, 90% in *E. coli,* and 83.3% in *P. mirabilis* isolates [4]. Antimicrobial resistance in GNB responsible for 45–70% of VAP, is a daily challenge to ICU physicians [59]. An epidemiological study on VAP patients reported 72.1% of MDR-GNB [53].

### Why has pneumonia etiology shifted to GNB?

Most of the people in the community, particularly in low-income countries, purchase cheap and freely available antibiotics from local drug stores and use them without a physician’s prescription, resulting in the ineffective killing of the causative agent, treatment failure, and the survival of resistant GNB, which increases the percentage of GNB resistant to drugs [60]. Poor infection control, inadequate antimicrobial stewardship, the limited vaccine coverage targeting GNB, their high burden in the hospital settings as a source of drug-resistant GNB spread to the community through hospital effluents, the difficult nature of acquiring resistance through transmissible genes, which act as a vector or reservoir of resistant genes, the increased comorbid conditions, the increased elderly populations, and the aggressive virulence determinants to cause severe disease are all reasons for the colonization of GNB [13, 61, 62].

### Risk factors for MDR-GNB

According to the studies report [63–65], MDR-GNB causing pneumonia can be acquired from the community or hospital setting with risk factors including prolonged hospital stay, prior MDR-GNB colonization or infection, high frequency of antibiotic resistance in the setting, ICU admission, mechanical ventilation, and surgical intervention. They are also common in the elderly population, patients with prior antibiotic use, those with underlying pulmonary diseases (such as chronic obstructive pulmonary disease and bronchiectasis), diabetes mellitus, immunosuppressive conditions (like HIV and malignancies), prior hospitalization, and chronic alcoholism [66–70]. In addition, enteral malnutrition and the use of carbapenem drugs are significant risk factors for PDR *A. baumannii*-induced VAP [71].

### Pathogenesis: the role of virulence determinants in transmission, colonization, adhesion, and invasion

Gram-negative bacterial pneumonia can be acquired through the aspiration of bacteria from parts of the upper respiratory tract or gastrointestinal tract (GIT), the inhalation of aerosols, hematogenous spread from distant sites such as the urinary tract, GIT, or lungs infected with *E. coli* and *P. aeruginosa* into the alveoli [72]. Additionally, bacterial translocation from the GIT has recently been known to be a mechanism of acquiring pneumonia [73]. Among these routes, aspiration is a common cause of HAP and CAP [74]. Approximately 45% of healthy adults aspirate oropharyngeal bacteria while sleeping, and abnormal swallowing of bacteria may also occur in people with depressed consciousness, respiratory tract instrumentation, and/or mechanically ventilated patients [75].

Bacterial translocation is a pathogenesis mechanism in which viable bacterial flora or enteric microorganisms of the GIT escape from the intestinal lumen through epithelial mucosa into the mesenteric lymph nodes and then, possibly, to the lung. The translocating organisms could be the cause of pneumonia, or they could cause changes in defense mechanisms that make it difficult for the host to clear a bacterial inoculum from the lungs. Patients with immunosuppression, cancer, or burns may experience this [76, 77].

The colonization of GNB substantially increases with previous use of antimicrobial agents in patients who have alcoholism, diabetes mellitus, pulmonary disease, or use of inhalation devices [78]. Adhesins, invasins, secretory molecules such as effectors and extracellular matrix, outer membrane vesicles, toxins, capsules, fimbriae, flagella, iron acquisition systems consisting of an outer membrane receptor, a periplasmic binding protein, and an inner membrane ABC transporter, and biofilm formation in GNB contribute to disease occurrence. Some of these processes, like adhesins, are found in chromosomes, whereas others, such as plasmids, are found in mobile genetic components. Siderophores, for example, are virulence factors that allow bacteria to adapt and live in a host by competing with normal flora for iron [79].

Adherence is aided by adhesions, which are bacterial surface structures that promote attachment to epithelial cells, pili, cilia, capsules, elastase production, host factors like surface proteins and polysaccharides, and environmental factors like pH and the presence of mucin in respiratory secretions. Malnutrition, severe illness, endotracheal intubation, and the postoperative state can all increase GNB adherence. Prolonged intubation causes the biofilm formation on the inner surface of the endotracheal tube, which contributes to pathogen persistence and treatment failure [80]. Invasion of GNB into the spaces between cells and adjacent alveoli via flagellar movement across the connecting pores causes neutrophil recruitment and cytokine release, resulting in immune system activation and inflammatory response. Due to lipopolysaccharide endotoxin, this inflammatory response is the primary cause of general respiratory symptoms such as fever, chills, fatigue, changes in blood pressure, and even shock. Neutrophils, bacteria, and fluid leaking from nearby blood vessels fill the alveoli, causing dyspnea due to impaired oxygen transportation. Severe pneumonia causes hypoxia, which leads to hyperventilation and death [81, 82].

### Challenges and major advancements in the diagnosis of pneumonia

Accurate pneumonia diagnosis is critical for determining the disease burden and developing effective treatment and prevention strategies. Currently, pneumonia is diagnosed based on the patient’s medical history and clinical signs and symptoms like cough, fever, purulent sputum, auscultation findings, acute pulmonary infiltrate, and dyspnea. Chest radiography and computed tomography scans are common radiographic imaging techniques used in the diagnosis of pneumonia [83]. It is impossible to determine the etiology of pneumonia based solely on clinical examination; instead, an optimal specimen must be obtained for laboratory identification of bacteria [84]. The inability to obtain good quality sputum due to contamination with normal respiratory flora, the good safety profile of transthoracic lung aspirates, and the difficulty of obtaining sputum in children and the elderly all posed challenges [85]. Routine culture, bacterial identification, and antimicrobial susceptibility testing need different specimens, require specimen treatment, poor detection rate, and long turnaround times up to 48–72 h.

Molecular diagnostic tests and/or nucleic acid detection tests have been used for the diagnosis of bacterial pneumonia over recent years. Rapid molecular detection of the pathogen can minimize the empirical use of broad-spectrum antibiotics in severe CAP, HAP, and VAP, but their interpretation is difficult due to differences in the local treatment guidelines and resistance genes, the discrepancy between genotype and phenotype, the ongoing discovery of new resistance mechanisms, and, as a result, the potential presence of unknown mechanisms, which may lead to false-negative results using molecular techniques [86]. A study by Kitsios et al. [87] about the etiologic diagnosis of bacterial pneumonia in mechanically ventilated patients reported that enhanced pathogen detection using microbial DNA sequencing can improve upon culture-based diagnosis, that sequencing profiles correlate with the host response, and offers substantial opportunity for individualized therapeutic targeting and antimicrobial stewardship. The multiplex polymerase chain reaction (M-PCR) has become useful for the rapid diagnosis of bacterial causes of pneumonia directly from the sputum and blood [88]. A recent prospective study used M-PCR to detect bacterial pathogens in 95 clinical bronchoalveolar lavages or plugged telescoping catheter samples from VAP patients and found that the M-PCR system had a global sensitivity of 80% and specificity of 99%. The sensitivity was better for GNB identification (90%) [89]. The Bio Fire Film Array Pneumonia Plus Panel is an FDA-cleared sample-to-answer assay that enables the detection of bacteria and antimicrobial resistance marker genes from sputum and bronchoalveolar lavage fluid [90]. It has a shorter turn-around-time than culture-based approaches, is more beneficial to a diverse set of patients with severe LRTI, such as severe CAP or VAP, who are routinely prescribed broad-spectrum empirical treatment, and can change antibiotic prescriptions in 40.7% of patients [91].

### Antimicrobial resistance mechanisms in GNB

Resistance to antimicrobial agents is increasing at both community and hospital levels, being especially relevant in the hospital settings in which changes in the hospital environment and strong selective pressure favor the selection, persistence, and maintenance of resistant, MDR (resistant to at least one agent in three or more antimicrobial classes), XDR (resistant to at least one agent in all but two or fewer antimicrobial classes), and even PDR strains (resistant to all the current groups of antibiotics for therapeutic use), causing antibiotic treatment failure, increased mortality, and morbidity, and having a significant impact on the cost of medical treatment and prevention of bacterial infections [92]. Antimicrobial resistance can be innate resistance by genes encoding inherent antibiotic resistance present in the bacteria, acquired resistance due to selective antibiotic pressure from the environment, or adaptative resistance that is a reflection of the ecological niche of the bacteria, including environmentally induced genetic changes. Mechanisms of antibiotic resistance include target alteration of the drug, the impermeability of the bacteria, bypassing the drug, efflux of the drug, biofilm formation, and genetically associated changes such as mutations and plasmid-mediated transfer of resistance genes [93, 94]. Additionally, GNB is resistant to antibiotics with an alteration in the outer membrane such as porin mutations, production of enzymes including beta-lactamase, carbapenemase, and aminoglycoside modifying enzymes (phosphorylating, adenylylating, and acetylating enzymes), and increased expression of the transmembrane efflux pump [95].

Alexander Fleming first discovered resistance in gram-negative species to beta-lactam antibiotics in 1929. Then now, resistance to beta-lactam drugs in GNB has frequently been studied and it is due to the production of beta-lactamase enzymes such as the active site serine beta-lactamases (classes A, C, and D) and the class B Metallo-beta-lactamases that use active site zinc ions to coordinate a nucleophilic hydroxide to mediate ring-opening [96]. The enzyme lactamase is formed in the periplasmic space, which inactivates the antibiotic after penetration into the bacterial organism and breaks the amide bond of the four-membered beta-lactam ring, deactivating the molecule’s antimicrobially active molecules through hydrolysis. The highly drug-resistant *P. aeruginosa*, *A. baumannii,* and *K. pneumoniae* in HAP and VAP patients encodes plasmid-mediated AmpC b-lactamases on their chromosomes that hydrolyze cephalosporins, monobactams, and cephamycins, as well as the expression of class A KPC b-lactamases, confer resistance to carbapenems [97]. Moreover, the loss of OprD associated with resistance to carbapenems such as imipenem and meropenem in *P. aeruginosa and i*ncreased production of drug efflux pump systems (*mex*), as part of either an acquired or intrinsic resistance repertoire, is capable of exporting various substrates from the periplasm of GNB to the surrounding environment before the action of the drug [98].

#### Biofilm-mediated resistance

A biofilm is an aggregate of microorganisms that are firmly attached to the biotic or abiotic surface, encased within an extracellular polymeric substance matrix, and that can show new characteristics to gene expression, protein synthesis, growth rate, and metabolic activities, thereby facilitating the anchorage to any surface irreversibly. The matrix confers antibiotic resistance through processes such as slow penetration of antibiotics, expression of chromosomally encoded resistant genes or development of persistent cells, changes in bacterial growth rate and metabolic activities, altered microenvironment due to depletion of nutrients and/or accumulation of waste substances that will antagonize the action of antibiotics, and even counteracting the host immunity [99]. In mechanically ventilated patients, biofilms are associated with endotracheal intubation, which acts as a reservoir for drug-resistant pathogens causing VAP such as *P. aeruginosa* and *A. baumannii* that persist in the hospital settings [100].

### Update on treatment options and promising future perspectives

Antibiotics are the treatment of choice for bacterial pneumonia, and the choice of antibiotic depends on the nature of pneumonia, the microorganism, and the immune status of the individual. In randomized double-blind trials, omadacycline, lefamulin, and delafloxacin were non-inferior to moxifloxacin for treating community-acquired bacterial pneumonia in adults [101–103]. The ongoing spread of antimicrobial resistance in pneumonia cases has made treating MDR-GNB empirically difficult [104]. A review by James et al. [23] about the novel antibiotics for the treatment of HAP and VAP caused by resistant GNB reported that ceftazidime has demonstrated noninferiority to meropenem against carbapenem-resistant Enterobacteriaceae (CRE) and ceftolozane against MDR *P. aeruginosa*. Recent noninferiority trials reported cefiderocol, ceftolozane/tazobactam, ceftazidime/avibactam, and imipenem/cilastatin/relebactam combinations as potential options in patients with MDR gram-negative nosocomial pneumonia, which showed non-inferior to high-dose, extended-infusion carbapenems such as meropenem in terms of clinical efficacy and all-cause mortality [105–108].

Aerosolized antibiotic therapy is already widely administered in ICUs during mechanical ventilation. A single-center, double-blind study on adjunctive therapy of ICU patients with confirmed MDR-GNB in VAP reported that aerosolized amikacin successfully eradicated existing MDR bacteria without inducing new resistance to amikacin or change in serum creatinine [109]. Colistin is a last resort therapy for infections caused by MDR-GNB, in particular *P. aeruginosa*, *A. baumannii*, and *K. pneumoniae* [110]. In critically ill patients with nosocomial pneumonia caused by MDR-GNB, including carbapenem-resistant strains, adjunctive nebulized colistin therapy provided non-inferior therapeutic efficacy to parenteral colistin therapy with lower clinical failure [111, 112].

The therapeutic potential of bacteriophages targeting MDR strains of GNB using animal models was evaluated in vitro and showed high infectivity of phages and multiple phage doses were required for effective treatment in vivo [113]. Novel treatment options such as PlyF307 lysine phage against MDR *A. baumannii*, VTCCBPA43 phage against virulent *K. pneumoniae*, PlyPa91 and vB_PaeP_PA01EW phages against *P. aeruginosa*, and Abp95 lytic *myoviridae* phage against multi-genotypes of carbapenem-resistant *A. baumannii* were demonstrated in mouse models [114–118]. Tridecaptins, a non-ribosomal lipopeptide, showed a selective bactericidal activity against the gram-negative version of the peptidoglycan precursor lipid II on the outer leaflet of the inner membrane and disrupts the proton-motive force [119].

In a mouse infection model, odilorhabdins also showed antimicrobial activity against GNB, including CRE, by binding to the small ribosomal subunit at a site not exploited by common antibiotics that induce miscoding, amino acid misincorporation, and bypass premature stop codons that interfere with protein synthesis [120]. Quorum sensing inhibitors can be applied along with other antibiotics such as Isobutyl-4, 5-Dihydroxy-2, 3-pentanedione (DPD) and phenyl-DPD with gentamicin and small molecules to fight biofilm-mediated drug resistance [60]. A recent study found that maipomycin A, a novel natural compound with promising anti-biofilm activity against pathogenic GNB, acts as a synergist to enhance colistin efficacy against *A. baumannii* [121]. In situations where antimicrobial treatment has been unsuccessful or where current therapies have caused resistance, iron-chelation therapy reduces the growth of MDR-GNB and potentiates antimicrobial strategies, particularly in HAP/VAP [122].

### GNB vaccine trials are currently underway

Even if vaccines against pneumonia were introduced in routine immunization programs, reaching for all people in low-income countries is a common challenge to tackling pneumonia. Although there is no licensed vaccine for clinical use against GNB, there is a new advance in vaccination. The polysaccharide capsules of *K. pneumoniae* have been previously targeted for developing therapeutics and vaccines for treating carbapenem-resistant *K. pneumoniae* infections [123]. A recent trial in a mouse model demonstrated the promising efficacy of new vaccine containing YidR recombinant protein to prevent *K. pneumoniae* disease [124]. The preclinical study reported that *K. pneumoniae* bioconjugates are immunogenic and effective, protecting mice against lethal infection from 2 hv*Kp* strains, NTUH K-2044 and ATCC 43816 [125].

Kumar et al. [126] evaluated the potential of recombinant FyuA of *K. pneumoniae* against lung infection in BALB/c mice and found that immunization generated both humoral and cell-mediated responses that conferred protection against the lethal dose of bacteria. A randomized clinical trial evaluated recombinant IC43 100 μg vaccination against potentially lethal *P. aeruginosa* infection in mechanically ventilated non-surgical ICU patients and found that it was both immunogenic and well-tolerated [127]. A live vaccine containing auxotrophic strain that lacks the key enzyme involved in D-glutamate biosynthesis, a structural component of the bacterial cell wall, confers mucosal immunity and protection against lethal pneumonia caused by *P. aeruginosa* [128]**.**

### Role of *Stenotrophomonas maltophilia* in pneumonia: an opportunistic GNB

*S. maltophilia* is an aerobic, non-fermenting, and environmental MDR-GNB, emerges in immunocompromised individuals and causes severe pneumonia [129]. It causes HAP in critically ill patients in the ICU due to its ability of biofilm formation and site adhesion in respiratory instruments and its intrinsic and acquired resistance to various antibiotics makes treatment difficult [130, 131]. In recent studies, the incidence of VAP due to *S. maltophilia* was 0.27 to 0.93% [131–133]. It causes severe hemorrhagic pneumonia with a reported mortality rate of 100% [134]. A study reported high mortality of *S. maltophilia* pneumonia in older cancer patients who used inappropriate antibiotic treatment [135]. Hemorrhagic pneumonia caused by *S. maltophilia* is a significant risk factor for mortality in patients with hematologic malignancy such as thrombocytopenia and prolonged neutropenia [136]. Currently, tigecycline is a promising alternative to trimethoprim-sulfamethoxazole and fluoroquinolones for treating VAP caused by *S. maltophilia,* but its resistance to available antibiotics has increased [137].

## Conclusion and recommendations

Worldwide, the prevalence of GNB among pneumonia patients is in the range of 49.7% to 95.3%. The predominant MDR-GNB in recently published studies causing pneumonia were *A. baumannii, K. pneumoniae, *and* P. aeruginosa, *with* A. baumannii *isolated particularly in VAP patients*.* The prevalence of MDR-GNB is higher in the elderly population, prior MDR-GNB infection, prolonged hospital stays, ICU admission, mechanical ventilation, surgical intervention, prior antibiotic use, comorbidity, chronic alcoholism, and enteral malnutrition. Although the resistance pattern of GNB varies from place to place, their resistance to commonly used antibiotics is almost similar in all studies across the country. In the majority reports of GNB, ampicillin, tetracyclines, and amoxicillin-clavulanic acid were highly resistant in CAP, whereas cephalosporins and carbapenems were in VAP. *S. maltophilia* became a severe cause of HAP in critically ill patients due to its ability of biofilm formation, site adhesion in respiratory equipment, and its intrinsic and acquired drug resistance mechanism. Microbial DNA sequencing, M-PCR, and the Bio Fire Film Array Pneumonia Plus Panel have been recently applied to detect bacterial pneumonia. Novel PCR-based techniques should be implemented for the early detection of drug-resistant genes to overcome the transmission of highly resistant genes between bacteria. Since there are increased MDR and PDR gram-negative strains, it makes the treatment more complicated, which may lead to high morbidity, economic losses, and mortality. To this end, newer, effective combination therapies with minimal clinical side effects, antibiotics against drug-resistant genes, antibiofilm agents, and vaccine approaches involving genetic vaccines or pathogen-specific lymphocytes, particularly for PDR strains, should be developed.

## Data Availability

All extracted data are freely available in the literature.

## Abbreviations

CAP: Community-acquired pneumonia
CRE: Carbapenem-resistant Enterobacteriaceae
ESBL: Extended-spectrum beta-lactamase
GIT: Gastrointestinal tract
GNB: Gram-negative bacteria
HAP: Hospital-acquired pneumonia
ICU: Intensive care unit
LRTIs: Lower respiratory tract infections
MDR: Multi-drug resistant
MDR-GNB: Multi-drug resistant gram-negative bacteria
MRSA: Methicillin-resistant *Staphylococcus aureus*
M-PCR: Multiplex polymerase chain reaction
PDR: Pan-drug resistant
VAP: Ventilator-associated pneumonia
XDR: Extensively drug-resistant

## References

[1] Mackenzie G (2016). The definition and classification of pneumonia. Pneumonia..

[2] Medical News Today (2019). What to know about bacterial pneumonia.

[3] Pahal P, Rajasurya V, Sharma S (2020). Typical Bacterial Pneumonia.

[4] Temesgen D, Bereded F, Derbie A, Biadglegne F (2019). Bacteriology of community acquired pneumonia in adult patients at Felege Hiwot referral hospital, Northwest Ethiopia: a cross-sectional study. Antimicrob Resist Infect Control.

[5] Shebl E, Gulick PG (2020). Nosocomial Pneumonia.

[6] Eshwara V, Mukhopadhyay C, Rello J (2020). Community-acquired bacterial pneumonia in adults: an update. Indian J Med Res.

[7] Roth GA, Abate D, Abate KH, Abay SM, Abbafati C, Abbasi N (2018). Global, regional, and national age-sex-specific mortality for 282 causes of death in 195 countries and territories, 1980–2017: a systematic analysis for the global burden of disease study 2017. Lancet.

[8] Cilloniz C, Martin-Loeches I, Garcia-Vidal C, San Jose A, Torres A (2016). Microbial etiology of pneumonia: epidemiology, diagnosis and resistance patterns. Int J Mol Sci.

[9] Miyashita N, Yamauchi Y (2018). Bacterial pneumonia in elderly Japanese populations. Jpn Clin Med.

[10] Ticona JH, Zaccone VM, McFarlane IM (2021). Community-acquired pneumonia: a focused review. Am J Med Case Rep.

[11] DerSarkissian C (2020). What is bacterial pneumonia?.

[12] Wong JL, Evans SE (2017). Bacterial pneumonia in patients with cancer: novel risk factors and management. Clin Chest Med.

[13] Henig O, Kaye KS (2017). Bacterial pneumonia in older adults. Infect Dis Clin.

[14] Hanada S, Pirzadeh M, Carver KY, Deng JC (2018). Respiratory viral infection-induced microbiome alterations and secondary bacterial pneumonia. Front Immunol.

[15] Marangu D, Zar HJ (2019). Childhood pneumonia in low-and-middle-income countries: an update. Paediatr Respir Rev.

[16] Jean S-S, Chang Y-C, Lin W-C, Lee W-S, Hsueh P-R, Hsu C-W (2020). Epidemiology, treatment, and prevention of nosocomial bacterial pneumonia. J Clin Med.

[17] del Valle-Mendoza J, Silva-Caso W, Cornejo-Tapia A, Orellana-Peralta F, Verne E, Ugarte C (2017). Molecular etiological profile of atypical bacterial pathogens, viruses and coinfections among infants and children with community acquired pneumonia admitted to a national hospital in Lima, Peru. BMC Res Notes.

[18] Chen G, Xu K, Sun F, Sun Y, Kong Z, Fang B (2020). Risk factors of multidrug-resistant Bacteria in lower respiratory tract infections: a systematic review and Meta-analysis. Can J Infect Dis Med Microbiol.

[19] Gao B, Li X, Yang F, Chen W, Zhao Y, Bai G (2019). Molecular epidemiology and risk factors of ventilator-associated pneumonia infection caused by carbapenem-resistant enterobacteriaceae. Front Pharmacol.

[20] Cillóniz C, Dominedò C, Torres A (2019). Multidrug resistant gram-negative bacteria in community-acquired pneumonia. Ann Update Intensive Care Emerg Med.

[21] Sader HS, Castanheira M, Mendes RE, Flamm RK (2018). Frequency and antimicrobial susceptibility of gram-negative bacteria isolated from patients with pneumonia hospitalized in ICUs of US medical centres (2015–17). J Antimicrob Chemother.

[22] Rouby J-J, Sole-Lleonart C, Rello J (2020). Ventilator-associated pneumonia caused by multidrug-resistant gram-negative bacteria: understanding nebulization of aminoglycosides and colistin. Intensive Care Med.

[23] Kidd JM, Kuti JL, Nicolau DP (2018). Novel pharmacotherapy for the treatment of hospital-acquired and ventilator-associated pneumonia caused by resistant gram-negative bacteria. Expert Opin Pharmacother.

[24] Cillóniz C, Dominedò C, Torres A (2019). Multidrug resistant gram-negative bacteria in community-acquired pneumonia. Crit Care.

[25] Roquilly A, Torres A, Villadangos J, Netea M, Dickson R, Becher B (2019). Pathophysiological role of respiratory dysbiosis in hospital-acquired pneumonia. Lancet Respir Med.

[26] Luan Y, Sun Y, Duan S, Zhao P, Bao Z (2018). Pathogenic bacterial profile and drug resistance analysis of community-acquired pneumonia in older outpatients with fever. J Int Med Res.

[27] Bjarnason A, Westin J, Lindh M, Andersson L-M, Kristinsson KG, Löve A (2018). Incidence, etiology, and outcomes of community-acquired pneumonia: a population-based study.

[28] Sattar SBA, Sharma S (2020). Bacterial Pneumonia.

[29] Malézieux-Picard A, Parent T, Roux X, Fassier T, Müller F, Prendki V (2021). Pneumonia prevention in the elderly patients: the other sides. Aging Clin Exp Res..

[30] Muthumbi E, Lowe BS, Muyodi C, Getambu E, Gleeson F, Scott JAG (2017). Risk factors for community-acquired pneumonia among adults in Kenya: a case–control study. Pneumonia..

[31] Jokinen J, Scott JAG (2010). Estimating the proportion of pneumonia attributable to pneumococcus in Kenyan adults: latent class analysis. Epidemiology.

[32] Onyedum CC, Chukwuka J (2011). Admission profile and management of community acquired pneumonia in Nigeria-5 year experience in a tertiary hospital. Respir Med.

[33] Markos Y, Dadi AF, Demisse AG, Ayanaw Habitu Y, Derseh BT, Debalkie G (2019). Determinants of under-five pneumonia at Gondar University hospital, Northwest Ethiopia: an unmatched case-control study. J Environ Public Health..

[34] Gordon S, Graham S (2006). Epidemiology of respiratory disease in Malawi. Malawi Med J.

[35] Aston SJ, Ho A, Jary H, Huwa J, Mitchell T, Ibitoye S (2019). Etiology and risk factors for mortality in an adult community-acquired pneumonia cohort in Malawi. Am J Respir Crit Care Med.

[36] Ojuawo OB, Desalu OO, Fawibe AE, Ojuawo AB, Aladesanmi AO, Opeyemi CM (2020). Clinical and microbiological profile of adult inpatients with community acquired pneumonia in Ilorin, north central, Nigeria. Afr Health Sci.

[37] Dessie T, Jemal M, Maru M, Tiruneh M (2021). Multiresistant bacterial pathogens causing bacterial pneumonia and analyses of potential risk factors from Northeast Ethiopia. Int J Microbiol.

[38] Assefa M, Tigabu A, Belachew T, Tessema B (2022). Bacterial profile, antimicrobial susceptibility patterns, and associated factors of community-acquired pneumonia among adult patients in Gondar, Northwest Ethiopia: a cross-sectional study. PLoS One.

[39] Kishimbo P, Sogone NM, Kalokola F, Mshana SE (2020). Prevalence of gram negative bacteria causing community acquired pneumonia among adults in Mwanza City, Tanzania. Pneumonia.

[40] Ibrahim A (2018). Bacterial etiology of community acquired pneumonia and their antimicrobial susceptibility in patients admitted to alshaab teaching hospital. Sudan Med Lab J..

[41] Torres A, Cillóniz C (2015). Clinical management of bacterial pneumonia.

[42] Pássaro L, Harbarth S, Landelle C (2016). Prevention of hospital-acquired pneumonia in non-ventilated adult patients: a narrative review. Antimicrob Resist Infect Control.

[43] Tassew SG, Alebachew Woldu M, Amogne Degu W, Shibeshi W (2020). Management of hospital-acquired infections among patients hospitalized at Zewditu memorial hospital, Addis Ababa, Ethiopia: a prospective cross-sectional study. PLoS One.

[44] Nusrat T, Akter N, Rahman NAA, Godman BD, Rozario DT, Haque M (2020). Antibiotic resistance and sensitivity pattern of Metallo-β-Lactamase Producing Gram-Negative Bacilli in ventilator-associated pneumonia in the intensive care unit of a public medical school hospital in Bangladesh. Hosp Pract.

[45] Sattar F, Quadros D, Olang P, Chokwe T (2018). Incidence of ventilator-associated pneumonia in the critical care unit at Kenyatta National Hospital, a public tertiary care hospital. East Afr Med J.

[46] Saied W, Mourvillier B, Cohen Y, Ruckly S, Reignier J, Marcotte G (2019). A comparison of the mortality risk associated with ventilator-acquired bacterial pneumonia and nonventilator ICU-acquired bacterial pneumonia. Crit Care Med.

[47] Cilloniza C, Dominedo C, Torresa A (2019). CURRENT OPINION an overview of guidelines for the management of hospital-acquired and ventilator-associated pneumonia caused by multidrug-resistant gram-negative bacteria. Curr Opin Infect Dis.

[48] Sader HS, Farrell DJ, Flamm RK, Jones RN (2014). Antimicrobial susceptibility of gram-negative organisms isolated from patients hospitalised with pneumonia in US and European hospitals: results from the SENTRY antimicrobial surveillance program, 2009–2012. Int J Antimicrob Agents.

[49] El-Nawawy A, Ramadan MA-F, Antonios MA-M, Arafa SA-F, Hamza E (2019). Bacteriologic profile and susceptibility pattern of mechanically ventilated paediatric patients with pneumonia. J Glob Antimicrob Resist.

[50] Mazloomirad F, Hasanzadeh S, Sharifi A, Nikbakht G, Roustaei N, Khoramrooz SS (2021). Identification and detection of pathogenic bacteria from patients with hospital-acquired pneumonia in southwestern Iran; evaluation of biofilm production and molecular typing of bacterial isolates. BMC Pulm Med.

[51] Feng D-Y, Zhou Y-Q, Zou X-L, Zhou M, Wu W-B, Chen X-X (2019). Factors influencing mortality in hospital-acquired pneumonia caused by gram-negative bacteria in China. J Infect Public Health.

[52] Bassetti M, Righi E, Vena A, Graziano E, Russo A, Peghin M (2018). Risk stratification and treatment of ICU-acquired pneumonia caused by multidrug-resistant/extensively drug-resistant/pandrug-resistant bacteria. Curr Opin Crit Care.

[53] Gupta R, Malik A, Rizvi M, Ahmed M, Singh A (2017). Epidemiology of multidrug-resistant gram-negative pathogens isolated from ventilator-associated pneumonia in ICU patients. J Glob Antimicrob Resist.

[54] Yehia FAA, Serry FK, Abdullatif H, M El-Ganiny A (2017). Prevalence and antimicrobial susceptibility of bacterial pathogens isolated from ventilator associated pneumonia (VAP) patients. Zagazig. J Pharm Sci.

[55] Ahsan AA, Barai L, Faruq MO, Fatema K, Ahmed F, Saha DK (2016). Antibiotic resistance pattern among bacteria causing ventilator associated pneumonia in an intensive care unit of Bangladesh. Bangladesh Crit Care J.

[56] Nguyen TT, Nguyen KT (2020). Hospital-acquired pneumonia in an intensive care unit in Vietnam: clinical characteristics and pathogenic bacteria. Pharmaceut Sci Asia.

[57] Nyawale HA (2020). Incidence, bacteria etiology and factors associated with ventilator associated pneumonia among patients on mechanical ventilator in intensive care units at tertiary hospitals.

[58] Gu W-J, Wang F, Tang L, Bakker J, Liu J-C (2014). Colistin for the treatment of ventilator-associated pneumonia caused by multidrug-resistant gram-negative bacteria: a systematic review and meta-analysis. Int J Antimicrob Agents.

[59] Ruppé É, Woerther P-L, Barbier F (2015). Mechanisms of antimicrobial resistance in gram-negative bacilli. Ann Intensive Care.

[60] Breijyeh Z, Jubeh B, Karaman R (2020). Resistance of gram-negative bacteria to current antibacterial agents and approaches to resolve it. Molecules..

[61] Asfaw T (2018). Review on hospital wastewater as a source of emerging drug resistance pathogens. J Res Environ Sci Toxicol.

[62] Cerceo E, Deitelzweig SB, Sherman BM, Amin AN (2016). Multidrug-resistant gram-negative bacterial infections in the hospital setting: overview, implications for clinical practice, and emerging treatment options. Microb Drug Resist.

[63] Maruyama T, Fujisawa T, Ishida T, Ito A, Oyamada Y, Fujimoto K (2019). A therapeutic strategy for all pneumonia patients: a 3-year prospective multicenter cohort study using risk factors for multidrug-resistant pathogens to select initial empiric therapy. Clin Infect Dis.

[64] Watkins RR, Van Duin D. Current trends in the treatment of pneumonia due to multidrug-resistant gram-negative bacteria. F1000Research. 2019;8. 10.12688/f1000research.16517.1 PMC6354321 30755795

[65] Rubio-Perez I, Martin-Perez E, Domingo-García D, Garcia-Olmo D (2017). Specific clinical profile and risk factors for mortality in general surgery patients with infections by multi-drug–resistant gram-negative bacteria. Surg Infect (Larchmt).

[66] Shindo Y, Ito R, Kobayashi D, Ando M, Ichikawa M, Shiraki A (2013). Risk factors for drug-resistant pathogens in community-acquired and healthcare-associated pneumonia. Am J Respir Crit Care Med.

[67] Prina E, Ranzani OT, Polverino E, Cillóniz C, Ferrer M, Fernandez L (2015). Risk factors associated with potentially antibiotic-resistant pathogens in community-acquired pneumonia. Ann Am Thorac Soc.

[68] Aliberti S, Cilloniz C, Chalmers JD, Zanaboni AM, Cosentini R, Tarsia P (2013). Multidrug-resistant pathogens in hospitalised patients coming from the community with pneumonia: a European perspective. Thorax..

[69] Eugenin EA (2013). Community-acquired pneumonia infections by Acinetobacter baumannii: how does alcohol impact the antimicrobial functions of macrophages?. Virulence..

[70] Inghammar M, Borand L, Goyet S, Rammaert B, Te V, Lorn Try P (2018). Community-acquired pneumonia and gram-negative bacilli in Cambodia—incidence, risk factors and clinical characteristics. Trans R Soc Trop Med Hyg.

[71] Yun Z, Hong Z, Dengchuan Z, Afang Z, Jiabin L. Risk factor analysis of pan-drug resistant *Acinetobacter baumannii*-induced ventilator-associated pneumonia in ICU. Indian J Pharm Sci. 2020:8–11.

[72] Teramoto SYK, Hizawa N (2015). Update on the pathogenesis and management of pneumonia in the elderly-roles of aspiration pneumonia. Respir Investig.

[73] Wang Y-h (2021). Current progress of research on intestinal bacterial translocation. Microb Pathog.

[74] Seki M (2020). Emerging the notion and definition of NHCAP: what is the NHCAP? Why aspiration pneumonia is important in NHCAP? Aspiration pneumonia.

[75] Sasegbon A, Hamdy S (2017). The anatomy and physiology of normal and abnormal swallowing in oropharyngeal dysphagia. Neurogastroenterol Motil.

[76] Nagpal R, Yadav H (2017). Bacterial translocation from the gut to the distant organs: an overview. Ann Nutr Metab.

[77] da Cruz DG, de Magalhães RF, Padilha GA, da Silva MC, Braga CL, Silva AR (2021). Impact of positive biphasic pressure during low and high inspiratory efforts in *Pseudomonas aeruginosa*-induced pneumonia. PLoS One.

[78] de Carvalho Baptista IM, Martinho FC, Nascimento GG, da Rocha Santos CE, do Prado RF, Valera MC (2018). Colonization of oropharynx and lower respiratory tract in critical patients: risk of ventilator-associated pneumonia. Arch Oral Biol.

[79] Cepas V, Soto SM (2020). Relationship between virulence and resistance among gram-negative bacteria. Antibiotics..

[80] Viswanathan P, Suneeva S, Rathinam P (2015). Quorum sensing in pathogenesis and virulence. Quorum sensing vs quorum quenching: a battle with no end in sight.

[81] Méndez R, Menéndez R, Cillóniz C, Amara-Elori I, Amaro R, González P (2018). Initial inflammatory profile in community-acquired pneumonia depends on time since onset of symptoms. Am J Respir Crit Care Med.

[82] Redinger J, Albert T (2020). Approach to dyspnea. chalk talks in internal medicine.

[83] Franquet T (2018). Imaging of community-acquired pneumonia. J Thorac Imaging.

[84] Noviello S, Huang DB (2019). The basics and the advancements in diagnosis of bacterial lower respiratory tract infections. Diagnostics..

[85] Claassen CC, Keenan WJ (2019). Challenging the “culture” of the tracheal aspirate. NeoReviews..

[86] Gadsby NJ, Russell CD, McHugh MP, Mark H, Conway Morris A, Laurenson IF (2016). Comprehensive molecular testing for respiratory pathogens in community-acquired pneumonia. Clin Infect Dis.

[87] Kitsios GD, Fitch A, Manatakis DV, Rapport SF, Li K, Qin S (2018). Respiratory microbiome profiling for etiologic diagnosis of pneumonia in mechanically ventilated patients. Front Microbiol.

[88] Akter S, Shamsuzzaman S, Jahan F (2014). Community acquired bacterial pneumonia: aetiology, laboratory detection and antibiotic susceptibility pattern. Malays J Pathol.

[89] Peiffer-Smadja N, Bouadma L, Mathy V, Allouche K, Patrier J, Reboul M (2020). Performance and impact of a multiplex PCR in ICU patients with ventilator-associated pneumonia or ventilated hospital-acquired pneumonia. Crit Care.

[90] Murphy CN, Fowler R, Balada-Llasat JM, Carroll A, Stone H, Akerele O (2020). Multicenter evaluation of the BioFire FilmArray pneumonia/pneumonia plus panel for detection and quantification of agents of lower respiratory tract infection. J Clin Microbiol.

[91] Edin A, Eilers H, Allard A (2020). Evaluation of the Biofire Filmarray pneumonia panel plus for lower respiratory tract infections. Infect Dis.

[92] Acharya KP, Wilson RT (2019). Antimicrobial resistance in Nepal. Front Med.

[93] Qi L, Li H, Zhang C, Liang B, Li J, Wang L (2016). Relationship between antibiotic resistance, biofilm formation, and biofilm-specific resistance in *Acinetobacter baumannii*. Front Microbiol.

[94] Arzanlou M, Chai WC, Venter H (2017). Intrinsic, adaptive and acquired antimicrobial resistance in gram-negative bacteria. Essays Biochem.

[95] Eichenberger EM, Thaden JT (2019). Epidemiology and mechanisms of resistance of extensively drug resistant gram-negative bacteria. Antibiotics..

[96] Öztürk H, Ozkirimli E, Özgür A (2015). Classification of beta-lactamases and penicillin binding proteins using ligand-centric network models. PLoS One.

[97] Bandić-Pavlović D, Zah-Bogović T, Žižek M, Bielen L, Bratić V, Hrabač P (2020). Gram-negative bacteria as causative agents of ventilator-associated pneumonia and their respective resistance mechanisms. J Chemother.

[98] Bonomo RA (2017). β-Lactamases: a focus on current challenges. Cold Spring Harb Perspect Med.

[99] Dumaru R, Baral R, Shrestha LB (2019). Study of biofilm formation and antibiotic resistance pattern of gram-negative Bacilli among the clinical isolates at BPKIHS. Dharan BMC Res Notes.

[100] Baidya S, Sharma S, Mishra SK, Kattel HP, Parajuli K, Sherchand JB (2021). Biofilm formation by pathogens causing ventilator-associated pneumonia at intensive care units in a tertiary care hospital: an armor for refuge. Biomed Res Int..

[101] Stets R, Popescu M, Gonong JR, Mitha I, Nseir W, Madej A (2019). Omadacycline for community-acquired bacterial pneumonia. N Engl J Med.

[102] File TM, Goldberg L, Das A, Sweeney C, Saviski J, Gelone SP (2019). Efficacy and safety of intravenous-to-oral lefamulin, a pleuromutilin antibiotic, for the treatment of community-acquired bacterial pneumonia: the phase III lefamulin evaluation against pneumonia (LEAP 1) trial. Clin Infect Dis.

[103] McCurdy S, Keedy K, Lawrence L, Nenninger A, Sheets A, Quintas M (2020). Efficacy of delafloxacin versus moxifloxacin against bacterial respiratory pathogens in adults with community-acquired bacterial pneumonia (CABP): microbiology results from the delafloxacin phase 3 CABP trial. Antimicrob Agents Chemother.

[104] Vardakas KZ, Trigkidis K, Falagas M (2017). Fluoroquinolones or macrolides in combination with β-lactams in adult patients hospitalized with community acquired pneumonia: a systematic review and meta-analysis. Clin Microbiol Infect.

[105] Wunderink RG, Matsunaga Y, Ariyasu M, Clevenbergh P, Echols R, Kaye KS (2021). Cefiderocol versus high-dose, extended-infusion meropenem for the treatment of gram-negative nosocomial pneumonia (APEKS-NP): a randomised, double-blind, phase 3, non-inferiority trial. Lancet Infect Dis.

[106] Torres A, Rank D, Melnick D, Rekeda L, Chen X, Riccobene T (2019). Randomized trial of ceftazidime-avibactam vs meropenem for treatment of hospital-acquired and ventilator-associated bacterial pneumonia (REPROVE): analyses per US FDA–specified end points.

[107] Titov I, Wunderink RG, Roquilly A, Rodríguez Gonzalez D, David-Wang A, Boucher HW, et al. A randomized, double-blind, multicenter trial comparing efficacy and safety of imipenem/cilastatin/relebactam versus piperacillin/tazobactam in adults with hospital-acquired or ventilator-associated bacterial pneumonia (RESTORE-IMI 2 Study). Clin Infect Dis. 2021;73(11):e4539–48. 10.1093/cid/ciaa803 PMC8662781 32785589

[108] Liscio JL, Mahoney MV, Hirsch EB (2015). Ceftolozane/tazobactam and ceftazidime/avibactam: two novel β-lactam/β-lactamase inhibitor combination agents for the treatment of resistant gram-negative bacterial infections. Int J Antimicrob Agents.

[109] Chang Liu Y-TZ, Peng Z-Y, Zhou Q, Hu B, Zhou H, Li J-G (2017). Aerosolized amikacin as adjunctive therapy of ventilator-associated pneumonia caused by multidrug-resistant gram-negative bacteria: a single-center randomized controlled trial. Chin Med J (Engl).

[110] Aygun F, Aygun FD, Varol F, Durak C, Cokugraş H, Camcioglu Y (2019). Can nebulised Colistin therapy improve outcomes in critically ill children with multi-drug resistant gram-negative bacterial pneumonia?. Antibiotics..

[111] Feng JY, Peng CK, Sheu CC, Lin YC, Chan MC, Wang SH (2021). Efficacy of adjunctive nebulized colistin in critically ill patients with nosocomial carbapenem-resistant Gram-negative bacterial pneumonia: a multi-centre observational study. Clin Microbiol Infect..

[112] Abdellatif S, Trifi A, Daly F, Mahjoub K, Nasri R, Lakhal SB (2016). Efficacy and toxicity of aerosolised colistin in ventilator-associated pneumonia: a prospective, randomised trial. Ann Intensive Care.

[113] Manohar P, Nachimuthu R, Lopes BS (2018). The therapeutic potential of bacteriophages targeting gram-negative bacteria using galleria mellonella infection model. BMC Microbiol.

[114] Lood R, Winer BY, Pelzek AJ, Diez-Martinez R, Thandar M, Euler CW (2015). Novel phage lysin capable of killing the multidrug-resistant gram-negative bacterium Acinetobacter baumannii in a mouse bacteremia model. Antimicrob Agents Chemother.

[115] Anand T, Virmani N, Kumar S, Mohanty AK, Pavulraj S, Bera BC (2020). Phage therapy for treatment of virulent *Klebsiella pneumoniae* infection in a mouse model. J Glob Antimicrob Resist.

[116] Raz A, Serrano A, Hernandez A, Euler CW, Fischetti VA (2019). Isolation of phage lysins that effectively kill *Pseudomonas aeruginosa* in mouse models of lung and skin infection. Antimicrob Agents Chemother.

[117] Huang L, Huang S, Jiang L, Tan J, Yan X, Gou C (2021). Characterization and sequencing of a novel phage Abp95 against multi-genotypes of Carbapenem-resistant *Acinetobacter baumannii*.

[118] Zhang Y, Meng B, Wei X, Li Y, Wang X, Zheng Y (2021). Evaluation of phage therapy for pulmonary infection of mouse by liquid aerosol-exposure *Pseudomonas aeruginosa*. Infect Drug Resist.

[119] Bann SJ, Ballantine RD, Cochrane SA (2021). The tridecaptins: non-ribosomal peptides that selectively target gram-negative bacteria. RSC Med Chem.

[120] Pantel L, Florin T, Dobosz-Bartoszek M, Racine E, Sarciaux M, Serri M (2018). Odilorhabdins, antibacterial agents that cause miscoding by binding at a new ribosomal site. Mol Cell.

[121] Zhang J, Liang X, Zhang S, Song Z, Wang C, Xu Y, Maipomycin A (2021). A novel natural compound with promising anti-biofilm activity against gram-negative pathogenic bacteria. Front Microbiol.

[122] Islam S, Chisti MJ, Ahmed M, Anwar N, Lehmann C (2019). Bacterial resistance in pneumonia in developing countries—a role for Iron chelation. Trop Med Infect Dis.

[123] Opoku-Temeng CKS, DeLeo FR (2019). *Klebsiella pneumoniae* capsule polysaccharide as a target for therapeutics and vaccines. Comput Struct Biotechnol J.

[124] Rodrigues MX, Yang Y, de Souza Meira EB, do Carmo Silva J, Bicalho RC (2020). Development and evaluation of a new recombinant protein vaccine (YidR) against *Klebsiella pneumoniae* infection. Vaccine..

[125] Feldman MF, Bridwell AEM, Scott NE, Vinogradov E, McKee SR, Chavez SM (2019). A promising bioconjugate vaccine against hypervirulent *Klebsiella pneumoniae*. Proc Natl Acad Sci.

[126] Kumar A, Harjai K, Chhibber S (2020). Early cytokine response to lethal challenge of *Klebsiella pneumoniae* averted the prognosis of pneumonia in FyuA immunized mice. Microb Pathog.

[127] Adlbrecht C, Wurm R, Depuydt P, Spapen H, Lorente JA, Staudinger T (2020). Efficacy, immunogenicity, and safety of IC43 recombinant *Pseudomonas aeruginosa* vaccine in mechanically ventilated intensive care patients—a randomized clinical trial. Crit Care.

[128] Cabral MP, Correia A, Vilanova M, Gärtner F, Moscoso M, García P (2020). A live auxotrophic vaccine confers mucosal immunity and protection against lethal pneumonia caused by *Pseudomonas aeruginosa*. PLoS Pathog.

[129] Gajdács M, Urbán E (2019). Prevalence and antibiotic resistance of *Stenotrophomonas maltophilia* in respiratory tract samples: a 10-year epidemiological snapshot. Health Serv Res Manag Epidemiol.

[130] Shah MD, Coe KE, El Boghdadly Z, Wardlow LC, Dela-Pena JC, Stevenson KB (2019). Efficacy of combination therapy versus monotherapy in the treatment of *Stenotrophomonas maltophilia* pneumonia. J Antimicrob Chemother.

[131] Guerci P, Bellut H, Mokhtari M, Gaudefroy J, Mongardon N, Charpentier C (2019). Outcomes of Stenotrophomonas maltophilia hospital-acquired pneumonia in intensive care unit: a nationwide retrospective study. Crit Care.

[132] Saied WI, Merceron S, Schwebel C, Le Monnier A, Oziel J, Garrouste-Orgeas M (2020). Ventilator-associated pneumonia due to *Stenotrophomonas maltophilia*: risk factors and outcome. J Infect.

[133] Canivet C, Teysseyre L, Aujoulat T, Caron M, Nativel M, Miltgen G (2022). Risk factors for a first episode of ventilator-associated pneumonia caused by *Stenotrophomonas maltophilia*.

[134] Imoto W, Kaneko Y, Yamada K, Kuwabara G, Yamairi K, Shibata W (2020). A mouse model of rapidly progressive fatal haemorrhagic pneumonia caused by *Stenotrophomonas maltophilia*. J Glob Antimicrob Resist.

[135] Velázquez-Acosta C, Zarco-Márquez S, Jiménez-Andrade MC, Volkow-Fernández P, Cornejo-Juárez P (2018). *Stenotrophomonas maltophilia* bacteremia and pneumonia at a tertiary-care oncology center: a review of 16 years. Support Care Cancer.

[136] Kim S-H, Cha MK, Kang C-I, Ko J-H, Huh K, Cho SY (2019). Pathogenic significance of hemorrhagic pneumonia in hematologic malignancy patients with *Stenotrophomonas maltophilia* bacteremia: clinical and microbiological analysis. Eur J Clin Microbiol Infect Dis.

[137] Zha L, Zhang D, Pan L, Ren Z, Li X, Zou Y (2021). Tigecycline in the treatment of ventilator-associated pneumonia due to *Stenotrophomonas maltophilia:* a multicenter retrospective cohort study. Infect Dis Ther.

